# Supplementary material for: Risk Factors Contributing to Reinfection by SARS-CoV-2: A Systematic Review
Source: Adv Respir Med. 2023 Dec 6;91(6):560–70. doi: 10.3390/arm91060041 (PMC10740414; doi:10.3390/arm91060041)
Supplement: Supplementary file 1 [file arm-91-00041-s001.zip › arm-2728899-supplementary.pdf]

Supplementary Material

Table S1.

**Search strategy**
**PUBMED**
("sars cov 2"[MeSH Terms] OR "sars cov 2"[All Fields] OR "covid"[All Fields] OR "covid 19"[MeSH Terms] OR "covid 19"[All Fields] OR ("sars cov 2"[MeSH Terms] OR "sars cov 2"[All Fields] OR "sars cov 2"[All Fields]) OR ("covid 19"[MeSH Terms] OR "covid 19"[All Fields] OR "coronavirus disease 2019"[All Fields]) OR ("sars cov 2"[MeSH Terms] OR "sars cov 2"[All Fields] OR "ncov"[All Fields])) AND ("reinfect"[All Fields] OR "reinfecting"[All Fields] OR "reinfection"[MeSH Terms] OR "reinfection"[All Fields] OR "reinfected"[All Fields] OR "reinfections"[All Fields] OR "reinfects"[All Fields] OR ("latent infection"[MeSH Terms] OR ("latent"[All Fields] AND "infection"[All Fields]) OR "latent infection"[All Fields] OR ("reactivated"[All Fields] AND "infection"[All Fields]) OR "reactivated infection"[All Fields]) OR (("second"[All Fields] OR "seconds"[All Fields]) AND ("episode"[All Fields] OR "episodes"[All Fields] OR "episodic"[All Fields] OR "episodically"[All Fields])))
**SCOPUS**
TITLE-ABS-KEY (coronavir* OR "corona virus" OR "corona pandemic" OR betacoronavir* OR covid19 OR covid OR ncov OR"CoV2"OR coV2 OR sarscoV2 OR sarS2 OR 2019ncov OR "novel CoV") OR TITLE-ABS-KEY(sars AND cov) AND (TITLE-ABS-KEY("severe acute respiratory" OR pneumonia* OR infection OR "respiratory infectious disease")) AND (TITLE-ABS-KEY(reinfection*OR "secondary infection")) AND (LIMIT-TO (PUBYEAR , 2021) OR LIMIT-TO (PUBYEAR , 2020)) AND(LIMIT-TO(DOCTYPE , "ar"))

Table S2. JBI assessment results of studies

**Country**	**Publication date**	**Author**	**1. Were the criteria for inclusion in the sample clearly defined?**	**2. Were the study subjects and the setting described in detail?**	**3. Was the exposure measured in a valid and reliable way?**	**4. Were objective, standard criteria used for measurement of the condition?**	**5. Were confounding factors identified?**	**6. Were strategies to deal with confounding factors stated?**	**7. Were the outcomes measured in a valid and reliable way?**	**8. Was appropriate statistical analysis used?**	**Total**
Italy	May-22	Sacco C, Petrone D, Del Manso M, et al.[24]	1	0	1	1	1	0	1	1	**6**
Italy	May-22	Flacco ME, Soldato G, Acuti Martellucci C, et al.[25]	1	0	1	1	1	1	1	0	**6**

Saudi Arabia	April 2022	Shaheen NA, Sambas R, Alenezi M, Alharbi NK, Aldibasi O, Bosaeed M [26]	1	1	1	1	1	0	1	1	**7**
Sweden	April 2022	Nordström P, Ballin M, Nordström A [27]	1	0	1	1	1	0	1	1	**6**
USA	August 2021	Slezak J, Bruxvoort K, Fischer H, Broder B, Ackerson B, Tartof S [28]	1	0	1	1	1	1	1	1	**7**
England	April 2022	Mensah AA, Lacy J, Stowe J, et al.[29]	1	1	1	1	1	1	1	1	**8**
China	December 2020	Yao MQ, Zheng QX, Xu J, et al. [30]	1	0	1	1	1	1	1	0	**6**

South Africa	March 2022	Pulliam JRC, van Schalkwyk C, Govender N, et al. [31]	1	1	1	1	1	1	1	1	**8**
Bahrain (Middle East)	August 2022	Almadhi M, Alsayyad AS, Conroy R, Atkin S, Awadhi AA, Al-Tawfiq JA, AlQahtani M [3]	1	0	1	1	0	0	1	1	**5**
Saudi Arabia	July 2022	Al-Otaiby M, et al.[32]	1	1	1	1	1	1	1	1	**8**
Mexico	April 2021	Murillo-Zamora E, Mendoza-Cano O, Delgado-Enciso I, Hernandez-Suarez CM [33]	1	1	1	1	0	1	1	1	**7**

Spain	January 2022	Sánchez-Varela N, Cinza-Sanjurjo S, Portela-Romero M [35]	1	1	1	1	0	0	1	1	**6**
China	May-20	Hu R, Jiang Z, Gao H, et al. [36]	1	0	1	1	1	1	1	0	**6**
Republic of Cyprus	March 2022	Quattrocchi A, Tsioutis C, Demetriou A, et al. [37]	1	1	1	1	0	1	1	1	**7**
USA	August 2021	Cavanaugh AM, Spicer KB, Thoroughman D, Glick C, Winter K[38]	1	1	1	1	1	0	1	1	**7**

Serbia	July 2022	Medić S, Anastassopoulou C, Lozanov-Crvenković Z, et al. [39]	1	1	1	1	0	1	1	1	**7**
Austria	February 2021	Pilz S, Chakeri A, Ioannidis JP, et al. [40]	1	1	1	1	1	1	1	0	**7**
Brazil	February 2021	Adrielle Dos Santos L, Filho PGG, Silva AMF, et al. [41]	1	1	1	1	0	1	1	1	**7**
Sweden	January 2020	Havervall S, Ng H, Jernbom Falk A, et al.[42]	1	0	1	1	1	0	1	0	**5**

India	January 2022	Nisha B, Dakshinamoorthy K, Padmanaban P, Jain T, Neelavarnan M.[43]	1	1	1	1	0	1	1	1	**7**
USA	May-22	Levin-Rector A, Firestein L, McGibbon E, et al. [44]	1	1	1	1	1	0	1	1	**7**
Several countries	August 2021	Sotoodeh Ghorbani S, Taherpour N, Bayat S, Ghajari H, Mohseni P, Hashemi Nazari SS [8]	1	0	1	1	0	1	1	1	**6**

France	March 2022	Nguyen NN, Houhamdi L, Hoang VT, et al. [45]	1	1	1	1	0	1	1	1	**7**
England	March 2022	Mensah AA, Campbell H, Stowe J, et al.[46]	1	0	1	1	1	1	1	0	**6**
Mexico	June 2022	Ochoa-Hein E, Leal-Morán PE, Nava-Guzmán KA, et al.[47]	1	1	1	1	0	0	1	1	**6**

Table S2: Newcastle–Ottawa Scale (NOS) Risk of Bias Assessment Results

**Study ID**	**Selection**			**Comparability***	**Outcome**		**Total (7⋆)**
	Representativeness of exposed cohort (⋆)	Selection of non-exposed cohort (⋆)	Ascertainment of exposure (⋆)	(⋆⋆)	Assessment of outcome (⋆)	Adequacy of follow up (⋆)	
Sacco C, Petrone D, Del Manso M, et al. [24]	*	*	-	⋆ ⋆	*	-	⋆ ⋆ ⋆ ⋆ ⋆ (5)
Flacco ME, Soldato G, Acuti Martellucci C, et al.[25]	*	*	-	⋆ ⋆	*	*	⋆ ⋆ ⋆ ⋆ ⋆ ⋆ (6)
Shaheen NA, Sambas R, Alenezi M, Alharbi NK, Aldibasi O, Bosaeed M[26]	-	*	*	⋆ ⋆	*	-	⋆ ⋆ ⋆ ⋆ ⋆ (5)
Nordström P, Ballin M, Nordström A[27]	*	*	-	* -	*	-	⋆ ⋆ ⋆ ⋆ (4)
Slezak J, Bruxvoort K, Fischer H, Broder B, Ackerson B,	*	*	-	⋆ ⋆	*	-	⋆ ⋆ ⋆ ⋆ ⋆ (5)

Tartof S [28]							
Mensah AA, Lacy J, Stowe J, et al.[29]	*	*	-	★ ★	*	*	★ ★ ★ ★ ★ ★ (6)
Yao MQ, Zheng QX, Xu J, et al.[30]	*	*	*	★ ★	*	-	★ ★ ★ ★ ★ ★ (6)
Pulliam JRC, van Schalkwyk C, Govender N, et al.[31]	-	*	*	★ ★	*	-	★ ★ ★ ★ ★ (5)
Almadhi M, Alsayyad AS, Conroy R, Atkin S, Awadhi AA, Al-Tawfiq JA, AlQahtani M [3]	*	*	*	- ★	*	-	★ ★ ★ ★ ★ (5)
Al-Otaiby M, et al.[32]	*	*	-	★ ★	*	*	★ ★ ★ ★ ★ ★ (6)
Murillo-Zamora E, Mendoza-Cano O, Delgado-Enciso I, Hernandez-Suarez CM [33]	*	*	-	★ ★	*	*	★ ★ ★ ★ ★ ★ (6)
Garduño-Orbe B, Sánchez-	*	*	-	★ ★	*	-	★ ★ ★ ★ ★ (5)

Rebolledo JM, Cortés-Rafael M, et al.[34]							
Sánchez-Varela N, Cinza-Sanjurjo S, Portela-Romero M [35]	*	*	-	★★	*	*	★★★★★★ (6)
Hu R, Jiang Z, Gao H, et al.[36]	-	*	*	★★	*	-	★★★★★ (5)
Quattrocchi A, Tsioutis C, Demetriou A, et al.[37]	*	*	-	★★	*	-	★★★★★ (5)
Cavanaugh AM, Spicer KB, Thoroughman D, Glick C, Winter K[38]	*	*	-	★★	*	-	★★★★★ (5)
Medić S, Anastassopoulou C, Lozanov-Crvenković Z, et al.[39]	*	*	-	* -	*	*	★★★★★ (5)
Pilz S, Chakeri A, Ioannidis JP, et al.[40]	*	*	*	★★	*	-	★★★★★★ (6)

Adrielle Dos Santos L, Filho PGG, Silva AMF, et al.[41]	-	*	*	⋆⋆	*	-	⋆ ⋆ ⋆ ⋆ ⋆ (5)
Havervall S, Ng H, Jernbom Falk A, et al.[42]	*	*	*	⋆⋆	*	-	⋆⋆⋆⋆⋆⋆ (6)
Nisha B, Dakshinamoorthy K, Padmanaban P, Jain T, Neelavarnan M.[43]	*	*	-	⋆⋆	*	*	⋆⋆⋆⋆⋆⋆ (6)
Levin-Rector A, Firestein L, McGibbon E, et al.[44]	*	*	-	- ⋆	*	*	⋆ ⋆ ⋆ ⋆ ⋆ (5)
Sotoodeh Ghorbani S, Taherpour N, Bayat S, Ghajari H, Mohseni P, Hashemi Nazari SS[8]	*	*	-	⋆⋆	*	-	⋆ ⋆ ⋆ ⋆ ⋆ (5)
Nguyen NN, Houhamdi L,	*	*	-	⋆⋆	*	*	⋆⋆⋆⋆⋆⋆ (6)

Hoang VT, et al.[45]							
Mensah AA, Campbell H, Stowe J, et al.[46]	-	*	*	⋆⋆	*	-	⋆⋆⋆⋆⋆ (5)
Ochoa-Hein E, Leal-Morán PE, Nava-Guzmán KA, et al.[47]	*	*	-	⋆⋆	*	-	⋆⋆⋆⋆⋆ (5)
Jang EJ, Choe YJ, Yun GW, et al.[48]	*	*	-	⋆⋆	*	-	⋆⋆⋆⋆⋆ (5)
